# A postgraduate curriculum for integrated care: a qualitative exploration of trainee paediatricians and general practitioners’ experiences

**DOI:** 10.1186/s12909-018-1420-y

**Published:** 2019-01-07

**Authors:** Ann Griffin, Laura Knight, Alex McKeown, Charlotte Cliffe, Arun Arora, Paul Crampton

**Affiliations:** 1 Research Department of Medical Education, UCL Medical School, Royal Free Hospital, Room GF/664, London, NW3 2PF UK; 2 Department of Psychiatry, University of Oxford, Warneford Hospital, Oxford, OX3 7JX UK; 3 Manchester University, Oxford Rd, Manchester, M13 9PL UK; 4 Research Department of Medical Education, UCL Medical School, The Directorate, 74 Huntley Street, London, WC1E 6AU UK

**Keywords:** Integrated care curriculum, Postgraduate, Medical education, Experiential learning, Qualitative, Leadership

## Abstract

**Background:**

Integrated care unites funding, administrative, organisational, service delivery and clinical levels to create connectivity, alignment and collaboration within and between care delivery and prevention sectors. It aims to improve efficiency by avoiding unnecessary duplication of resources. Consequently, implementing integrated care is increasingly important; however, there are many barriers and how we teach healthcare practitioners to work across systems is under-researched. This paper explores an innovative educational curriculum, the Programme for Integrated Child Health (PICH).

**Methods:**

The PICH involved an experiential learning approach supported by taught sessions on specific issues relevant to integrated care. A qualitative study was conducted by interviewing 23 participants using semi-structured one-to-one interviews. Participants included trainees (general practice, paediatrics) and programme mentors. Data was thematically analysed.

**Results:**

Results are coded under three main themes: integrated care curriculum components, perceptions of a curriculum addressing integrated care and organisational change, and personal and professional learning. The data highlights the importance of real-world projects, utilising healthcare data, and considering patient perspectives to understand and develop integrated practices. Trainees received guidance from mentors but, more crucially learnt from, with, and about one another. They learnt about the context in which GPs and paediatricians work and developed a deeper understanding through which integrated services could be meaningfully developed.

**Conclusions:**

This study explored participants’ experiences and can be taken forward by educationalists to design curricula to better prepare healthcare practitioners to work collaboratively. The emergence of integrated care brings about challenges for traditional pedagogical approaches as learners have to re-align their discipline-specific approaches with evolving healthcare structures. PICH demonstrated that trainees acquired knowledge through real-word projects and experiential learning; and that this facilitated integration, empowering doctors to become leaders of organisational change. However, there are also many challenges of implementing integrated curricula which need to be addressed, including breaking down professional silos and integrating resourceful healthcare. This study begins to demonstrate the ability of an integrated curriculum to support trainees to work collaboratively, but further work is needed to develop the wider efficacy of the programme incorporating other professional groups, and to assess its longer term impact.

**Electronic supplementary material:**

The online version of this article (10.1186/s12909-018-1420-y) contains supplementary material, which is available to authorized users.

## Background

### The mandate for integrated care

The justification for enhancing the delivery of integrated care is largely predicated on the requirement to optimise the use of healthcare resources [1, 2]. Integrated care, an approach which incorporates funding, administrative, organisational, service delivery and clinical levels to create connectivity, alignment and collaboration within and between care delivery and prevention sectors is seen as critical in delivering modern healthcare [3]. Inefficiencies in the system pervade the whole healthcare economy, from macro-level structures through to micro-systems at the level of care delivery. Improving the economic efficiency of healthcare is mandated because of the increased demands put upon the UK National Health Service (NHS) – such as longer life expectancy, increases in chronic disease and complex health problems, and the need to provide more effective preventative and social care [4]. Integrated care, while varying in its specific type and intensity, broadly requires that healthcare provision is better coordinated and more patient-centred. The Health and Social Care Act [5] provided the political steer and raised the integration agenda as a priority. The aim of the Act was to improve coordination between primary and secondary care and between health and social care. This was thought to reduce hospital admissions and increase the amount of care that can be provided in the community (thereby reducing pressure on frontline healthcare resources, and improving patients’ experience of negotiating their health and instances of illness). By better integrating systems and processes, the argument presented is that this will reduce overlap and fragmentation of healthcare provision, thereby allowing smoother and more structured patient care [1, 2, 6].

There are significant challenges in moving the integrated care agenda into practice. Co-producing new systems of care in collaboration with a diverse range of stakeholders (including patients) and the concomitant reorganisation of governance structures and communication channels is fundamental to success. Collaborative practices within the workplace is underpinned by health workers who have received effective training in interprofessional education [7]. Managing demand, increasing capacity in certain areas (for example primary care), and strong leadership are essential considerations when constructing new integrated care services [1, 5]. Professional territorialisation, ‘turf wars’, and a lack of clarity about how we educate healthcare professionals to work in an integrated way only exacerbate existing blocks [8]. Research suggests that there are problems with mutual understanding and communication as Specialists complain about inadequate information and unnecessary referrals, whilst GPs have expressed dissatisfaction with lack of information, failure to take account of important psycho-social information and delays in communication; the two branches of the profession have such different core values that lack of understanding is inevitable [9].

### Developing an integrated care curriculum

To date there is little research on how to effectively teach integrated care to postgraduate doctors through a formal curriculum; however, six papers were identified on integrated care training programmes in or across specific medical domains [10–15]. Three of these were programmes which integrated primary care with secondary care for psychological rather than physical problems [10–12]. The other three report on less domain-specific programmes, focusing on integrated approaches to teaching medical ethics [13]; complementary and alternative medicine [14]; and interprofessional education generally, in training for doctors [15]. Methods of teaching integrated care in these studies included a range of approaches including lectures and seminars as well as integrated care placements. The teaching methods are guided by the same set of key principles which underpin the integrated care approach, namely: the need to ensure that medical trainees receive a well-rounded and balanced education, involving programmes where doctors from different specialisms and allied health professionals are learning together.

At the undergraduate level, integrated care learning has grown in prominence in recent years through the development of longitudinal integrated clerkships (LIC) [16, 17]. LICs are typically for a prolonged period of time (12 weeks plus) in which medical students follow patients through the healthcare system and fulfil the core curriculum. The benefits of these placements have been encouraging and as such the expansion continues across many different medical schools and contexts across the world [18, 19]. However, despite a wealth of evidence for undergraduate training there is little at the postgraduate level. Here there is fragmentation across the programmes as learners are taught discipline-specific knowledge and skills as they try to become a member of their chosen profession. Herein lies the difficulty of educating doctors to become both a specialist and also generalists who can work effectively across systems. The current study is focused on how disciplines and professions work together to provide integrated care and how these can be embedded into formal curriculum, rather than consisting in the informal and hidden curriculum as is more commonplace amongst postgraduate training programmes [20].

### Research questions

The study aimed to explore the essential prerequisites for an integrated care curriculum for newly qualified and trainee general practitioners and paediatric trainees and how this could effectively be implemented into modern healthcare systems and change. In particular we sought to explore:

1. What are trainees’ and mentors’ perceptions of integrated care and organisational change?
2. How is an integrated care curriculum experienced by postgraduate doctors, and what factors contribute to its effectiveness?
3. What outcomes do participants experience when undertaking a postgraduate integrated care curriculum?

## Methods

### Study setting

The study was set in London, UK. Participants were recruited from two cohorts of the Programme for Integrated Child Health (PICH). PICH was launched in 2014, hosted by the London School of Paediatrics, with the aim of preparing paediatric and GP trainees for new ways of working in the delivery of child health in new models of care. Trainees volunteered to take part in this year-long programme which was not part of formal postgraduate training.

### Curriculum design: Programme for Integrated Child Health (PICH)

The year-long curriculum consisted of acquisitive (e.g. ten evening sessions) and participatory (e.g. workplace-based service improvement project) approaches to learning [21]. The seminars covered three main themes: understanding and shaping integrated care services mindful of the patient experience and using co-production as an approach to service improvement; using data to drive change and developing services by understanding how clinicians work across boundaries, commissioning and leadership. The combined approach enabled learners to acquire key knowledge and facts relevant to integrated care and negotiate their own understanding of the concepts as they were transferred and applied to practice. Alongside the formally taught sessions on integrated health care, trainees were encouraged to contribute to new ways of working through the design and implementation of integrated care projects in their own clinical setting. They received senior level support from local educational supervisors as well as an allocated mentor with experience of integrated care. Full details of the programme are available at http://www.pich.org.uk.

### Participants

The study population comprised of trainees and mentors from the first two cohorts of PICH. Cohorts 1 and 2 differed with respect to the trainee group. In its first year PICH was run solely for trainee paediatricians and was expanded to include GPs in the second year. However, mentors for both cohorts were drawn from paediatrics and general practice.

### Data collection

Given the interactive participatory nature of the development of the PICH course, we decided to use an interpretivist approach [22] to frame our study so that we could explore how participants made use of this curriculum within their working practice. In order to gather data which could explore the full divergence of such learning approaches a qualitative methodology was deemed the most appropriate. The developmental nature of the course also aligned itself better to explorative research approaches as the participants’ experiences were unknown, and therefore we did not know what outcomes to explore.

A semi-structured interview schedule (see Additional file 1) allowed the research team to explore the concepts deemed important to the study whilst allowing respondents to contribute flexibly about their experience of the PICH programme. Participants were recruited initially via an email to all mentors and trainees from cohorts 1 and 2. Interviews were conducted either in person or by telephone, according to the interviewee’s preferences. Interviews were conducted by one researcher (AM) in order to provide consistency. Interviews were audio recorded for accuracy and transcribed professionally.

### Data analysis

A coding scheme was developed inductively (with meaning generated from the data) as well as deductively (to answer the questions posed by the research) [23]. The interviews were independently coded by four team members (AG, CC, AA & AM) using QSR NVIVO 11©. Coders were experienced medical education researchers, and represented perspectives from both insiders (e.g. GP, junior doctor) and outsiders (e.g. non-clinical researchers, academics). An initial coding scheme was developed based on four team members analysing the same five transcripts. The team members independently coded the transcripts, followed by a discussion to compare and contrast understandings which were used to devise the first iteration of the coding framework. Thereafter, the remaining transcripts were distributed between three of the team members for coding. Once this second round of coding had been done, coding was again compared and inter-coder reliability tests were performed to ensure coding consistency. Throughout the analysis stage the research team met repeatedly and worked closely in ensuring the development of a shared understanding of the meaning of the data. Inter-rater reliability of the initial thematic analysis was found to be 90% or above. Any discrepancies in coding themes or deficiencies in inter-coder reliability were discussed and a final coding framework agreed. Post data coding, three researchers (AG, PC & LK) analysed the data and interpreted the findings in relation to the literature.

## Results

### Participant characteristics

Following ethical approval by the UCL Joint Research Office, interviews took place with 15 trainees and 8 mentors, 23 PICH participants in total. Eighteen of the participants were paediatricians and five were from general practice. Four trainees were from the first cohort and 11 from the second cohort. Interviews were conducted between August 2016 and January 2017, the average length of the interviews was 30 min but this ranged between 14 and 46 min. The breakdown of participant demographics (*participant number; trainee/mentor; GP/paediatrician; cohort 1/cohort 2) are detailed in the table below (Table 1).

**Table 1:** Participant demographics

Participant type: Trainee (T)/mentor(M)	Medical specialism: General practice (GP)/Paediatrician (P)	Cohort number: 1 = 1st year 2 = 2nd year	Participant identifier
T	GP	Cohort 2	P1TGP2*
M	GP	Cohort 1 & 2	P2MGP
T	Paediatrician	Cohort 1	P3TP1
T	Paediatrician	Cohort 2	P4TP2
M	Paediatrician	Cohort 1 & 2	P5MP
T	Paediatrician	Cohort 2	P6TP2
M	Paediatrician	Cohort 1 & 2	P7MP
M	Paediatrician	Cohort 1 & 2	P8MP
T	Paediatrician	Cohort 2	P9TP2
T	Paediatrician	Cohort 1	P10TP1
M	Paediatrician	Cohort 1 & 2	P11MP
T	Paediatrician	Cohort 2	P12TP2
T	Paediatrician	Cohort 2	P13TP2
M	GP	Cohort 1 & 2	P14MGP
T	GP	Cohort 2	P15TGP2
M	Paediatrician	Cohort 1 & 2	P16MP
T	Paediatrician	Cohort 2	P17TP2
M	Paediatrician	Cohort 1 & 2	P18MP
T	GP	Cohort 2	P19TGP2
T	Paediatrician	Cohort 1	P20TP1
T	Paediatrician	Cohort 1	P21TP2
T	Paediatrician	Cohort 2	P22TP2
T	Paediatrician	Cohort 2	P23TP2

### Themes

We identified three core themes related to (1) integrated care curriculum components, (2) perceptions of a curriculum addressing integrated care and organisational change, and (3) personal and professional learning.

### Theme one: Integrated care curriculum components

The format of the integrated curriculum highlighted the strengths and challenges of how it was experienced by trainees and enacted in practice. The curriculum was heavily focused on integrating experiential learning with traditional pedagogical approaches covering seminars, individual projects and mentoring. The induction session, the project website, the mentoring scheme, and the monthly seminars were all largely evaluated positively.

#### Seminars

The monthly seminars were reported as one of the most important parts of the PICH, since these provided regular opportunities for trainees to seek feedback and advice on their projects from mentors and peers, to present their ongoing work, to share knowledge, and to hear presentations from invited speakers about different aspects of integrated child health. Trainees and mentors equally valued the seminars; however, for some there was difficulty in having enough time to attend the course regularly:

They’ve [mentors] split it so that the mentors and mentees can meet and talk in small groups, but then hear a more formal presentation, so it’s very well formatted and incredibly valuable I think for people like myself to then take the extra time to go. P16MP

I don’t think it’s the downfall of the PICH Project. I think it’s more to do with the fact our day jobs allowing us to do more… I can’t get study leave to go and do the clinics*.* P23TP2

#### Mentoring

The mentoring system was arranged in such a way that each trainee was attached to a senior GP or paediatrician with expertise in integrated care. Their stated role was to provide general assistance and support, and also to assist with trainees’ personal projects over the course of the year. On the whole, trainees from both cohorts found the mentoring extremely rewarding, with P12TP2 describing it as ‘*a real highlight of the year’*. Where criticisms were raised about the mentoring they were primarily about lack of access to them during and after the course and this was particularly noticeable for those trainees who were not able to complete their projects during the PICH year. The mentors’ view of the purpose of the system was ambitious but straightforward, in that they wanted to provide trainees with new skills, but also identify individuals who can be ‘*standard bearers*’ for integrated care in future, beyond the life of PICH:

I think it was probably very good for me…you know they’re [mentors] all quite inspiring high powered people, which is a bit intimidating to begin with. And I didn’t see her that often, but I do feel that when I did see her she usually had something you know…something that really kind of started to nudge me in the right direction*.* P21TP2

…what we’re [mentors] trying to do is twofold, is to equip these people with some skills to do it and some skills to change things where they are, but what we also really want is within each group to try and inspire a few…three or four each time who are really going to go on and lead on this in the future, so not only just do their own little bit, but also really take the kind of baton forward for integrated child health*.* P8MP

#### Individual projects

The projects were mostly positively evaluated throughout both cohorts; a typical response was voiced by the trainee below, who found that the project gave them access to resources which they did not know how to access previously.

I suppose the thing that’s had the most impact on me is being able to access data, like national data, and being able to look at it in more detail knowing where to go to get information and statistics for each CCG*.* P1TGP2

Criticisms appeared to mostly relate to misunderstandings about what the projects were meant to achieve in terms of tangible, completed outputs at the end of the programme. Those trainees who voiced unhappiness about their projects often felt disillusioned about what they had achieved or how it could be useful. Mentors, however, were able to recognise such learning opportunities and facilitate greater trainee reflection on the pragmatic value of the project.

I’ve finished it and I’m not really sure…what was the point of my whole project. I mean I’ve learnt a lot and that’s great and that’s wonderful, I’ve learnt great amounts, but I’ve now got a project I don’t really know what I’m supposed to do with it*.* P13TP2

Even if the projects don’t always turn out as extensive as some of the people engaged in the work think at the beginning, I think people reflecting on what they’ve learnt find that invaluable, because there’s nothing as powerful as a learning experience as doing it yourself*.* P18MP

Other trainees found that their learning had been valuable even in the absence of a clear and readily quantifiable end result. For example, one trainee suggested that the process of being immersed in the practice of facilitating integrated care provided their most important learning experience. This approach was reflected in the mentors’ accounts of the purposes of the trainees’ projects.

When they’re doing a project…they very quickly come up against you know obstacles, which is the reality of working in the NHS. And so rather than ending up with a sort of half-finished project, you know the obstacles should be the object of study, if you see what I mean, because that is in fact what you get in real life. If you have the skills to overcome them, you’re going to be very effective*.* P7MP

There was a strong emphasis on the educational value of the projects deriving from practical attempts at implementation and the development of problem-solving skills, rather than the production of a more conventional completed piece of written work. The mentor’s perspective above highlights the importance of mentors’ experience in identifying learning opportunities and considering the challenges that trainees are likely to encounter when undertaking practice-based projects.

### Theme two: Perceptions of a curriculum addressing integrated care and organisational change

For trainees and mentors, integrated care is understood to be core to community healthcare delivery; however, with so many factors involved there is uncertainty about how to embed the concept into practice. Through the individual project’s process of immersing trainees in the practice of facilitating integrated care, the curriculum provided the opportunity for participants to critically engage with how collaborative practice is currently enacted in the clinical workplace. Participants reported a number of issues relevant to the implementation of integrated care and the organisational change necessary for it. These issues were discussed throughout our interviews and highlighted the barriers to developing integrated care, how to maintain efficiency and effectiveness in integrated care, and how patient centeredness could be prioritised within integrated care.

#### Attitudes to integrated care

Trainees, mentors and programme leads held strongly positive views about the value of integrated care*.* The main beliefs expressed about integrated care were that it improved patient outcomes through more cost-efficient patient pathways; providing holistic care which was not “*necessarily a medical need*” (P22TP2) but which, through integration across primary and secondary care, created a “*joined up system*” (P5MP).

I think fundamentally because it creates a system that responds, that is designed around the patient, so instead of the patient doing the running around and joining up, the system is joined up and therefore the patient’s needs are met in a holistic way, in an efficient way with resources used as effectively as possible*.* P5MP

#### System and organisational change

While trainees, mentors and programme leads held strongly positive views about the *value* of integrated care, they also felt strongly that it was challenging to translate the idea into practice. Participants’ beliefs about the reason for these challenges focussed on three key themes, each of which will now be discussed.

(i) Professional and structural barriers to implementing integrated care

Participants perceived many obstacles relating to effectively implementing integrated care. They reported that barriers operated at both the macro and micro levels. Individual (micro) level barriers included professionals with fixed beliefs or an unwillingness to change current ways of working, and participants described colleagues as too busy to attend clinics in the community or to work with other health professionals in different environments. Participants stated that the busy and stressful nature of their work meant that it became difficult to think innovatively and act on integrating care. However, it was systems (macro) level barriers that were most commonly noted. In particular, staff shortages and heavy service demands were reported to inhibit “*going out and doing some proactive care*” (P10TP1). There were also disciplinary barriers within professions (e.g. nursing) that provided resistance.

One of the trainees was telling me the other day that you know she was doing an asthma project, and you know one of the obstacles that she came across was you know that school nurses were not interested in collaborating with the asthma nurses*.* P7MP

The structural organisation of primary and secondary care was also described as an issue. The way GPs and hospitals are funded was highlighted as a particular problem, where money follows GP referrals such that if more patients are treated in the community there is a possible financial impact on hospitals. The structural challenges encountered when working between primary and secondary care were viewed as being a particular instance of an NHS-wide phenomenon of people working within established specialty-specific and organisational boundaries:

…integrated care keeps patients out of hospital and bringing patients into hospital is how hospitals get their income. P5MP

…we do an incredible amount of silo working and we do things a certain way because that’s the way they’ve always been done. And so trying to create change within NHS organisations seems to be incredibly challenging*.* P12TP2

Some participants talked about how barriers might develop during undergraduate training, believing that a lack of interprofessional education in the undergraduate curriculum may make it difficult to adopt integrated approaches to healthcare later in a clinician’s career. Accordingly, some participants felt that doctors did not always understand integrated care and how it could be implemented. Despite these difficulties, participants nonetheless felt that integrated care provided a logical framework for the efficient design and delivery of services.

…for the vast majority of the people they’ll train in a medical school or a nursing school – stay in one place and never get out of the four walls. That can’t be good for patient care… I think it’s terribly challenging though, it’s extremely challenging, because people at undergraduate level don’t get exposed enough*.* P16MP

…the barriers are the clinicians not understanding it or the concept…most departments at most hospitals wouldn’t even have one member of senior staff that would necessarily know what integrated care’s all about*.* P21TP2

(ii) Healthcare delivery is *in*efficient but evidence for change is challenging to produce

It was felt that the benefits of ‘integrated care’ were still under-evaluated. Participants articulated two opposing views of how clinicians who know about integrated care could make use of that knowledge in practice. One view was that these clinicians should drive this patient-centred process improvement forward to develop improvements in efficiency. The other, more passive, view was that clinicians will be equipped to accommodate or adapt to the arrival of macro-level changes in the organisation of the healthcare infrastructure, and or when they come:

I think it’s looking at how different parts of the health service interact. Looking at how to identify an issue, use data to back up that as an issue and then looking at how best to look how you might improve - how you might look at improving what - the issue that you’ve identified, using different tools. P9TP2

Much of healthcare is moving out from secondary care into primary care in the community. And because of that, because we’re trying to keep people out of hospital and manage them there, you have to have an integrated care project, there’s no other way of doing it safely and effectively for a patient. P14MGP

In either case, though particularly the former, participants were aware of the need to convince colleagues of the effectiveness of integration. They suggested that this was difficult to quantify and thus to adduce as evidence to others. However, there was evidence that even in the absence of ‘hard’ outcome data, doctors are aware of inefficiencies in the system that result in either gaps in treatment or duplication of services. Integrated care was seen as a means to iron out inefficiencies, to work across traditional organisational interfaces, exploring the best way to revise systems to improve efficiency and to dissolve structural barriers:

We are currently in a very evolving and changing NHS with very strange services and working together is going to be a way to improve patient outcomes and be more efficient, you know, saving resources when we can...there is a lot of wastage on appointments that are maybe not needed. P1TGP2

Integrated care is a good example of making sure that boundaries don’t become barriers… The porosity becomes extremely important, and this is a great example of trying to put some holes into the boundary to try and get people flowing across the boundary. P16MP

(iii) A lack of focus on patient-centred care

Many trainees and mentors felt that the patient journey is currently fragmented and disjointed, and participants highlighted the need for consistency and fluidity in care. The majority of the participants focused on the importance of ‘patient need’ providing the direction and drive for treatment decisions. Placing patients at the centre for requirement for integrated care was a recurring theme in the data analysis, and both mentors and trainees shared this view. P16MP gave the following explanation of why integration is important for patient care:

…there were two things that mattered - that the patients were satisfied and the professionals were satisfied that they were doing a good job. So integrated care is incredibly important because it’s a means to that end. And you can call it whatever you want – integrated care, connected care, community … doesn’t matter what you call it, all it is the joining up of people with an end in mind which is the best possible care, the most efficient care, the safest care, the most timely care to your patients.

In doing so, P16MP highlights that integrated care is important for from both the perspective of the patients, and the clinicians attempting to deliver it for them. Trainees also cited having a clear understanding of patients’ wishes as a means for delivering better care:

I think our patients dictate why it’s important…And they want, you know, joined up care, they want, you know, not there to be this massive kind of ‘here’s primary care, here’s secondary care, here’s tertiary care’ – they want a flow, they want to be looked after in a holistic manner… they’re our main concern*.* P15TGP2

The most powerful learning… is that it’s about patient experience, how you can just, by making little efforts and sitting down with patients talking through their experience…you actually truly can understand where they’re coming from and what’s actually important to them…you can then make a much more informed decision how to change and improve care*.* P3TP1

### Theme three: Personal and professional learning

Given the lack of formal education on integrated care in undergraduate and postgraduate education, understanding the impact of the curriculum on participants was important to understand its effectiveness. The trainees and mentors believed the programme was effective, particularly in the domains of clinical experience, personal development and leadership.

#### Leadership

PICH appeared to enable trainees to gain leadership skills through autonomously developing new ways to deliver integrated care. The realisation that leadership skills could be deployed in order to deliver integrated care beyond the individual doctor’s traditional clinical circle of influence stimulated some trainees to consider developing an interest in non-clinical roles:

…in my future career I can definitely see myself going into more clinical commissioning, and I think I’ll be much more able to have an eye for things like integrated healthcare. And that’s something I really want to be involved in, like as a project for… like as a roll-out either locally or nationally whatever, and I think that’s … it has more of an impact that way*.* P15TGP2

#### Personal development

Participants reported that PICH helped to broaden trainees’ and mentors’ understanding of practice and service provision. They also discovered that learning about integrated care could broaden a doctor’s perspective on their work. This was because they perceived integrated care to be ‘*boundary-less…unlimited*’, and thus supported an understanding of the ‘*bigger picture’*. Mentors’ reflections on their engagement with trainees were also illuminating, as they enabled more experienced clinicians to see situations and problems in their own practice afresh.

Participants reported that they gained confidence over the course of PICH. Two aspects appeared to be influential in this. One was asking for help from mentors and peers and receiving positive responses. The other aspect was the project work, which improved their confidence in handling data. PICH was also reported to enable trainees to understand themselves better by reflecting on their values and skills as a doctor. Several trainees reflected on how PICH had led them to think about their future practice. Some considered what they could do now to incorporate patient experiences more fully in their clinical practice whilst some considered future career choices:

[The programme has been]…hugely helpful to me as a doctor … has given me a real clarity of thought about what’s right about that approach, a real determination to really work hard to transform things to do something about it. P11MP

It confirms to me that you know it’s something that I really want to get involved in the future… because of service provision and commissioning, that’s something I’m really interested in*.* P15TGP2

I wouldn’t know about how to use the data or patient experience and involvement. It’s just something that we come across, but don’t actually necessarily get any training in, so that’s been really helpful. P9TP2

#### Clinical experience

Trainees frequently reported that they acquired clinical knowledge and that projects were an activity that promoted learning, even if they were not completed over the year trainees and trainers commented on their educational value. Mentors also reflected on how PICH has been beneficial in terms of their own clinical learning. Participants noted how learning from each other was an important affordance of the programme and not readily available in most clinical workplaces. In particular several remarked that the process of teaching clinical skills improved their own understanding of what they were delivering:

I’ve probably learnt that in terms of doing projects, the end outcome isn’t as important as I probably previously thought, and the journey … the learning point along that way probably are, and there’s a bit emphasis on that, that it doesn’t really matter if you get to the end or finished, but you’ve learnt and you’ve done something along the way, even if it’s not completely finished*.* P1TGP2

It reminds me about best practice, so actually in the same way that you know if you teach something you learn it really well*.* P8MP

I’ve been taught by the respiratory lead, I’ve been taught by the respiratory nurses. So in terms of my clinical skills, I think they’ve improved hugely in terms of managing asthma in paediatrics*.* P1TGP2

If you’re a secondary care paediatric trainee it’s important for you to be able to see what’s out there in the community … and vice versa for the GP trainees. But as an addition to that and more-so it’s an invaluable experience in terms of allowing trainees to train each other, so peer supported learning … in a new environment, with those that are experienced within it. And you don’t get that in training programmes at the moment, they’re very much caged into their own speciality. P14MGP

## Discussion

The findings reveal the usefulness of a postgraduate integrated curriculum while highlighting the strengths and difficulties of implementing such a concept in modern health systems (theme two), the key pedagogical components of an integrated care curriculum (theme one) and the personal and professional learning experienced by participants (theme three). Integrated curricula are becoming commonplace at the undergraduate level [16, 24] but postgraduate education has yet to embed this approach. This study builds on the existing literature which, like other developments, allowed learners to develop interprofessional knowledge and skills [10, 15, 25].

A prominent feature of the integrated curriculum was the emphasis on self-direction and learner autonomy which were captured in theme three, personal and professional learning. The requirement for prospective participants to demonstrate a degree of self-direction prior to joining the course, for example by identifying senior support within their hospital or practice, probably functioned to ensure that participants were indeed self-directed and motivated. Another central feature of the curriculum was the emphasis on experiential learning through the design and implementation of authentic workplace-based integrated care projects developing their clinical experience (theme one, individual projects). These projects required trainees to engage with data and develop ‘systems’ thinking in considering how to improve services for patients in ways that, in all probability, they would not otherwise have done. The problem-based and real-world orientation of the projects appeared to be essential to the identity of the PICH programme. Yet, key to the success of these projects was the provision of mentorship (see theme one), although some trainees struggled to regularly engage with their mentors outside of the seminars due to time pressures.

PICH is somewhat unconventional in emphasising ongoing skills development and personal development, rather than just the acquisition of a prescribed body of information. The learning environment was one of the successes of the PICH programme uncovered in theme one and two, pinpointing positive attitudes and addressing barriers. It generated great enthusiasm and passion and appeared to be extremely effective in raising morale. In theme one the data suggested that the seminars in particular engendered infectious feelings of positivity towards the goals and methods of the programme, and this was in some cases vitally important when set against a backdrop of severe and systemic difficulties in the NHS.

A final important finding identified in theme three was that learning was multidirectional – trainees and mentors both reported learning from each other and trainees reported learning significantly from their peers. Of particular note was the finding that most of the mentors reported having learnt from the trainees (see theme three: clinical experience); they appeared to derive value from reflecting on how trainees handled clinical situations that were familiar to the mentors, with the relative freshness that comes from having spent less time practising. These experiences appeared to help the mentors reflect on reasons behind their views or judgements, and thus to critically assess their own practice. There was, therefore, indication that PICH was able to promote personal professional development even amongst the most senior participants.

At the undergraduate level the impact of integrated curricula has been dominated by a mantra of patient centeredness extending to patient advocacy that is enabled through students experiencing patient continuity [16, 26]. Similarly in the current study, we found that patient centeredness was enriched by the curriculum as learners could begin to understand the challenges faced by patients in their navigation across community health services (theme two). This finding supports the literature to advance the view that a full understanding of a patient’s needs can only be achieved through adopting an integrated approach [10, 14, 15]. This can be formed by sharing the views of not only the patient, but also those of all the various specialist physicians whose input is relevant to determining the appropriate treatment or course of action. However, there were other benefits from PICH unreported in the literature which went beyond advocacy and related to leadership and how postgraduate trainees can begin to tackle and invoke systems change (theme three: leadership). As the NHS faces evolving healthcare structures the requirement for doctors to be able to react to such circumstances is critical to ensure safe patient care.

Despite healthcare systems requiring healthcare professionals to work collaboratively, there are very little formal curricula which tackle such issues and prepare learners to adopt this way of working. A main strength of this study is that it provides a qualitative in-depth insight into an integrated curriculum for postgraduate trainees. The main limitations of this research relate to the sample size and sampling approach, which was largely pre-determined via participation in the programme. Given the curriculum was voluntary the doctors who took part were likely to be a motivated group with a predisposition towards applying practices which create integrated health care pathways. This notion was substantiated by our finding that most participants had previous experience of integrated care, some of them through their involvement in the Learning Together Clinics which were a precursor to the PICH project. Nonetheless, in addition to identifying the impact of the programme on participants, we sought to identify the wide ranging views on how integrated healthcare can be incorporated into modern healthcare systems and what challenges are faced. The well-defined methodology allowed us to explore factors from both trainees and mentors perspectives to give a well-rounded conceptualisation of how integrated curricula can fit into postgraduate training.

This pilot programme has been shown to be effective in encouraging integrated healthcare practices which are pivotal to ensure efficiency throughout healthcare delivery. However, as it is highly likely that course participants in these early stages represent the early adopters, further research is needed into how universally effective it can be. This will enable the development of insight into how the principles revealed in the PICH evaluation could be applied to mainstream postgraduate training. The evaluation gives tentative cause for optimism that, given the right conditions and educational environment, training can be delivered to junior doctors that will enable them to innovate in their own professional environments, creatively applying the principles required for improving patient care and efficiency by joining up services in a way that is sustainable and meets the contemporary challenges of healthcare in the UK.

## Conclusion

This study explored the experiences of participants and mentors of an integrated care curriculum and can be taken forward by educationalists to design curricula to better prepare healthcare practitioners to work collaboratively. PICH was perceived to be well run, worthwhile, and provided the desired benefits in terms of education and learning about how integrated care can be delivered. The projects gave participants an enhanced understanding of how using real data could influence traditional systems: an authentic problem-based approach. It also provided a sense of autonomy, enabling them to craft something of personal and professional relevance, to innovate and shape their own clinical environment. While participants did talk about learning clinical knowledge and skills in a speciality to which they would not necessarily have exposure, the vast majority of their talk was directed towards their own personal development: gaining confidence, independence, forming networks, tools for individual reflection and application. An important finding from the interviews, with both trainees and mentors, was that the course appeared to be successful in delivering tools for leadership too. Participants acquired skills to take forward integrated care initiatives; ready to enact change as ‘leaders’ of integrated care for the future. While there are opportunities for improvements PICH provides an innovative example of a formal curriculum at the postgraduate level that can be further developed to ensure more efficient healthcare delivery.

## Additional file

Additional file 1: Copy of Interview Questions. (DOCX 13 kb)

## Abbreviations

GP: General Practitioner
LIC: Longitudinal Integrated Clerkship
NHS: National Health Service
PICH: Programme for integrated Child Health
