# Supplementary material for: A postgraduate curriculum for integrated care: a qualitative exploration of trainee paediatricians and general practitioners’ experiences
Source: BMC Med Educ. 2019 Jan 7;19:8. doi: 10.1186/s12909-018-1420-y (PMC6322273; doi:10.1186/s12909-018-1420-y)
Supplement: Supplementary file 1 — Copy of Interview Questions. (DOCX 13 kb) [file 12909_2018_1420_MOESM1_ESM.docx]

**Additional file 1-**

**Interview Questions**

**Background**

1. Firstly, please could you tell me whether you are from:
   1. Cohort 1 or 2?
   2. Trainee or trainer?
   3. GP or paediatrician?
2. How did you become involved in PICH?
3. Did you have any experience of integrated care or working with other medical specialities in this way prior to this program?

**The programme**

1. Please could you tell me about the PICH programme and course?
2. What do you think is / are the aim/s of PICH?
3. What aspects of the course did you feel worked well?
4. Which aspect, if any, has been the most educational / useful and why, e.g. your personal project, the CYP meetings?
5. What aspects of the program are working less well?
6. Do you think PICH has covered everything needed to enable the delivery of integrated care? If not, what was missing and could be improved upon?
7. Has participation in PICH surprised you in any way, and if so, how?
8. What have you learnt on PICH as a trainee or a trainer?

*Prompts (ensure cover of the following):*

1. *What did you learn about yourself?*
2. *About others?*
3. *About patient care?*
4. *About your organisation?*
5. How would you describe working with peers?
6. How would you describe working with trainers or trainees?

**Integrated care and working together**

1. Please reflect on working with clinicians from a different specialism, i.e. paediatrics or general practice:
   1. what have you learnt about the other specialism,
   2. what impact if any has it had on your understanding of your own?
2. Do you think interprofessional healthcare training is important, and if so why?
3. Do you think integrated care is important, and if so why?
4. Do you think there are any barriers to delivering integrated care? If so, what are they?
5. How do you think that participating in PIC H will impact on the way you work in the future?
6. What impact, if any, has PICH had on you as a clinician?

*Prompt: What have you learnt? Has PICH changed the way you work in a clinical context? If so, how?*
